# Perceived organizational support and family–school collaboration among kindergarten teachers: job satisfaction as a mediator and occupational commitment as a moderator

**DOI:** 10.3389/fpsyg.2026.1798098

**Published:** 2026-06-25

**Authors:** Tengteng Li, Yantao Shi, Lianhai Shi

**Affiliations:** 1 Faculty of Education, Guangxi Normal University, Guilin, China; 2 College of Innovation and Entrepreneurship, Guangxi Minzu University, Nanning, China; 3 Teaching and Resaerch Department of University Governance, National Academy of Education Administration, Beijing, China

**Keywords:** family–school collaboration, job satisfaction, kindergarten teachers, occupational commitment, perceived organizational support

## Abstract

Perceived organizational support has been found to correlate with family–school collaboration, yet the mediating and moderating mechanisms underlying this association—particularly under different levels of occupational commitment—remain insufficiently understood. The present study examined whether job satisfaction mediates the relationship between perceived organizational support and family–school collaboration, and whether kindergarten teachers’ occupational commitment moderates the direct and indirect pathways linking perceived organizational support to family–school collaboration via job satisfaction. Participants were 1,312 kindergarten teachers in China, recruited through convenience sampling. They completed self-report measures of perceived organizational support, family–school collaboration, job satisfaction, and occupational commitment. Results indicated that perceived organizational support was positively associated with family–school collaboration, and that job satisfaction partially mediated this association. Moderated mediation analyses further showed that occupational commitment moderated the path from perceived organizational support to job satisfaction. Specifically, the positive association between perceived organizational support and job satisfaction was stronger among teachers with higher occupational commitment. These findings elucidate the mechanisms through which perceived organizational support relates to family–school collaboration and advance understanding of the mediating process and its conditional effects, thereby providing empirical evidence on when and why teachers are more likely to sustain high-quality family–school collaboration.

## 1 Introduction

Family–school collaboration in early childhood education refers to an educational model in which families and kindergartens jointly promote children’s growth and development through sustained cooperation and interaction (Smith et al., 2022; Epstein, 1995). Its primary aim is to meet children’s multidimensional developmental needs—cognitive, emotional, and social—thereby reflecting the collaborative, holistic, and developmental nature of early childhood education (Iruka et al., 2022; Lang et al., 2024). Empirical work indicates that partnership effectiveness is jointly shaped by communication quality, parental beliefs and involvement, and kindergarten-level organizational commitment (Shi and Zheng, 2025; Ogg et al., 2021). Put differently, high-quality communication helps reduce information asymmetry and strengthens goal alignment between home and school. Parents’ educational beliefs and level of involvement not only determine the intensity of investments in the home learning environment but also shape the proactivity and continuity of parent–teacher interactions, thereby providing children with more stable cognitive stimulation and emotional support (Fujisawa et al., 2025; Schock and Jeon, 2023; Lohndorf et al., 2021). At the organizational level, perceived organizational support influences the stability, reach, and implementation fidelity of family–school collaboration through institutionalized arrangements, collaboration platforms, and the provision of training resources (Snell et al., 2022; Silver and Coba-Rodriguez, 2025). Moreover, stronger family–school collaboration has been positively associated with children’s learning engagement (Tan et al., 2021), social skills and social competence (Liu et al., 2024), as well as psychological safety and a sense of belonging (Froiland, 2021).

Perceived organizational support (POS) refers to employees’ global beliefs concerning the extent to which the organization values their work contributions and cares about their well-being and development (Eisenberger et al., 1986). Organizational support theory posits that employees form POS based on organizational cues such as procedural justice, supervisor support, and rewards and job conditions; higher POS is expected to satisfy employees’ socioemotional needs and, through the norm of reciprocity, strengthen felt obligation toward the organization, enhance affective commitment, and reduce strain reactions and emotional exhaustion (Rhoades and Eisenberger, 2002; Kurtessis et al., 2017). Within the context of family–school partnerships, POS can be conceptualized as teachers’ subjective appraisal of the structural and resource-based supports that the kindergarten provides for home–school collaboration, including institutionalized procedures and delineated responsibilities, communication platforms and information infrastructure, training and coaching resources for communication and conflict management, as well as time/workload protections and recognition-based incentives (Smith et al., 2021). Prior research suggests that when teachers report higher POS, they are more likely to perceive family–school partnership tasks as feasible and to hold stronger competence beliefs, which in turn facilitates more proactive and higher-quality parent–teacher communication and goal alignment, enhances the stability and implementation fidelity of collaborative practices, and ultimately promotes overall partnership effectiveness (Wei and Zhang, 2025; Smith et al., 2021; Fujisawa et al., 2025).

Perceived organizational support (POS) is significantly associated with family–school collaboration. Drawing on organizational support theory (OST), POS reflects teachers’ global perceptions that their kindergarten values their work contributions and cares about their well-being (Wei and Zhang, 2025; Hsieh et al., 2022). When teachers perceive higher organizational support, their socioemotional needs are more likely to be fulfilled; consequently, they tend to develop stronger felt obligation toward the organization and are more willing to engage in supportive and cooperative behaviors that go beyond minimal role requirements (Eisenberger et al., 1986; Rhoades and Eisenberger, 2002; Kurtessis et al., 2017). Family–school collaboration requires teachers to engage in proactive communication, information sharing, facilitation of parental involvement, and conflict management. These practices typically entail additional time investments and substantial emotional labor, thereby increasing teachers’ reliance on a supportive organizational context. Accordingly, emerging evidence suggests that kindergartens can strengthen the stability, reach, and implementation fidelity of family–school collaboration by institutionalizing procedures, establishing communication and coordination platforms, providing training and coaching resources for parent communication and conflict management, and offering time/workload protections as well as recognition-based incentives (Ash et al., 2024; Badiuzzaman et al., 2025; Smith et al., 2025). Moreover, high-quality communication reduces information asymmetry and enhances goal alignment, which facilitates the formation and maintenance of collaborative partnerships (Azad et al., 2021; Berlinski et al., 2025). When schools actively support teachers’ parent-facing work, teachers are more likely to sustain frequent, responsive, and ongoing engagement with families, thereby promoting higher levels of family–school collaboration (Garbacz et al., 2025; Gershy and Katz, 2023).

Kindergarten teachers’ perceived organizational support (POS) permeates multiple components of daily care-and-education practices and exerts an important influence on their work attitudes and behavioral performance (Cai et al., 2025; Li et al., 2025). Recent empirical research on family–school collaboration and parent–teacher interactions in early childhood settings has further highlighted the facilitating role of POS in strengthening home–school partnerships. Specifically, prior studies have shown that POS is positively associated with family–school collaboration (Jung and Sheldon, 2020; Epstein et al., 2011), and that teachers who perceive higher levels of support are more likely to sustain more proactive, higher-quality, and more enduring communication and collaborative engagement with families (Smith et al., 2021). Collectively, this line of evidence suggests that POS constitutes a key organizational antecedent of family–school collaboration. Nevertheless, the psychological processes through which POS translates into stronger family–school collaboration—such as potential mediating and moderating mechanisms—remain insufficiently established. To address this gap, the present study proposes a moderated mediation model that examines job satisfaction as a mediator linking POS to family–school collaboration and tests occupational commitment as a boundary condition that moderates the relevant pathways. In doing so, the study aims to clarify both how and under what conditions POS more strongly promotes kindergarten teachers’ family–school collaboration.

### 1.1 The mediating effect of job satisfaction

Job satisfaction refers to kindergarten teachers’ overall sense of satisfaction with their childcare and educational work experiences, the support they receive for professional development, and the quality of the organizational environment (Schock and Jeon, 2021; Jeon and Wells, 2018). It reflects a positive appraisal of work-related meaning and value, affective pleasure, and the intention to remain in the profession. Organizational support theory posits that through ongoing interactions with their kindergarten, teachers develop a global belief about whether the organization values their contributions and cares about their well-being (Eisenberger et al., 1986; Eisenberger et al., 2020). Prior research suggests that when teachers perceive adequate guarantees in resource allocation, institutional support, and emotional care, their social–emotional needs are more readily fulfilled, thereby strengthening their sense of belonging, perceived value, and positive self-concept (Cai and Tang, 2021; Chen, 2025; Kong et al., 2026; Wator et al., 2025). These supportive cues further promote more favorable affective evaluations of the work context, which manifests as higher levels of job satisfaction (Li et al., 2025; Zhu et al., 2024). Moreover, higher perceived organizational support can activate reciprocity-based motivation, enhance affective commitment and organizational trust, and encourage teachers to adopt more constructive cognitive appraisals of managerial decisions and resource arrangements, thereby reducing perceived unfairness and threat and ultimately improving job satisfaction (Demir and İnandı, 2023; Ertuğrul and Dereli, 2023; Zhang et al., 2023).

Existing empirical evidence supports a positive association between perceived organizational support and teachers’ job satisfaction. Specifically, studies have shown that higher perceived organizational support is significantly related to higher levels of job satisfaction (Zhu et al., 2024; Chen, 2025), suggesting that organizational support can be conceptualized as a salient contextual antecedent of job satisfaction. In addition, research using kindergarten teachers as the target sample has examined the linkage between perceived organizational support and job satisfaction and provided convergent evidence for their positive relationship (Liu et al., 2023; Zhu et al., 2024). Collectively, these findings indicate that perceived organizational support is positively and significantly correlated with job satisfaction. Drawing on organizational support theory and the extant empirical literature, we propose the following hypothesis:

*H1a*: Perceived organizational support is positively and significantly associated with kindergarten teachers’ job satisfaction.

Job satisfaction exerts an important influence on family–school collaboration. According to the Job Demands–Resources (JD–R) model, teachers’ occupational functioning is shaped by the interplay between job demands and job resources (Admiraal and Røberg, 2023; Reintjes et al., 2025), and these conditions affect subsequent work attitudes and behavioral manifestations through two core pathways: the health-impairment process and the motivational process (Collie, 2023). Family–school collaboration constitutes a high-demand and sustained work task that requires teachers to invest additional time and energy and to engage in substantial emotional labor, communication and coordination, and conflict management (Carey and Sutton, 2024; Mukhlis et al., 2026). When kindergarten teachers have ample job resources, they are more likely to experience elevated work engagement and more positive work experiences, thereby developing favorable work attitudes (Carey and Sutton, 2024; Semelius Granevald et al., 2024). By contrast, when resources are insufficient under high demands, kindergarten teachers are more prone to cumulative stress and emotional exhaustion and may adopt “resource conservation” strategies—such as reducing the frequency of parent interactions and limiting the depth of communication—thereby weakening their behavioral investment in family–school collaboration and undermining interaction quality (Powers et al., 2025; Trauernicht et al., 2023; Wang et al., 2025).

Moreover, prior research indicates that job satisfaction reflects teachers’ overall positive appraisal of their work context and the availability of resources (Liu et al., 2023; Zhu et al., 2024). Higher job satisfaction is typically accompanied by stronger motivational states, which can facilitate sustained resource investment and continued participation in family–school collaboration activities (e.g., home–school communication, shared decision-making, and follow-up), ultimately enhancing implementation effectiveness (Yang et al., 2025; Zagkotas et al., 2024). Empirical studies further demonstrate a significant positive association between teachers’ job satisfaction and family–school collaboration, providing support for these theoretical expectations (Lutovac et al., 2024; Zagkotas et al., 2024). Based on the JD–R model and the extant empirical evidence, we propose the following hypothesis:

*H1b*: Kindergarten teachers’ job satisfaction is positively and significantly associated with family–school collaboration.

Building on the hypothesized positive association between perceived organizational support and job satisfaction, as well as the hypothesized positive association between job satisfaction and family–school collaboration, we further posit that job satisfaction serves as a significant mediator in the relationship between perceived organizational support and family–school collaboration. Although prior research has not yet directly tested this specific mediational pathway, extant evidence provides indirect support for this proposition. Specifically, studies have shown that job satisfaction can function as a key intervening mechanism linking family–school collaboration to various antecedent conditions, such that organizational-level supportive resources are translated into teachers’ greater collaborative investment, stronger sustained participation in home–school interactions, and higher-quality communication.

### 1.2 The moderating effect of occupational commitment

Occupational commitment refers to an overarching psychological orientation formed by individuals’ affective attachment to their profession, their intention to sustain investment in it, and their sense of normative responsibility. It is typically manifested as a relatively enduring motivational tendency to remain in the profession and continue contributing over time. Prior research indicates that occupational commitment is a core work-attitudinal correlate of family–school collaboration (Antony-Newman, 2024; Otero-Mayer et al., 2026). Teachers with higher occupational commitment are more likely to construe family–school collaboration as an integral component of their professional role (Anazia, 2025; Otero-Mayer et al., 2026). Although the significant association between occupational commitment and family–school collaboration has been supported empirically, research on the conditional and indirect roles of occupational commitment (e.g., moderating effects) remains comparatively limited. Accordingly, the present study proposes that occupational commitment moderates both the direct and indirect relationships between perceived organizational support and family–school collaboration.

The present study proposes that occupational commitment moderates the association between perceived organizational support (POS) and teachers’ job satisfaction. Conservation of Resources (COR) theory (Hobfoll, 1989, 2001) suggests that individuals are motivated to acquire, maintain, and protect valued resources at work, and that resource gains and losses constitute core mechanisms shaping affective experiences, attitudinal evaluations, and subsequent behavioral regulation (Demerouti, 2025; Hlado et al., 2025; Liu et al., 2025). Within this framework, POS can be conceptualized as a salient job resource, reflecting teachers’ global belief that the organization values their contributions and cares about their well-being (Gou et al., 2025; Kong et al., 2026). Empirical evidence further indicates that higher POS is conducive to the fulfillment of teachers’ social–emotional needs, strengthens feelings of being valued and belonging, and in turn enhances job satisfaction (Kong and Wang, 2024; Zhu et al., 2024). Occupational commitment, in contrast, represents a relatively stable personal motivational resource (Amlaku et al., 2025; Su, 2024), manifested in teachers’ affective attachment to the profession, willingness to sustain investment, and sense of normative responsibility (Özdemir et al., 2024). We argue that teachers with higher occupational commitment are more likely to derive their job attitudes from intrinsic motivation and the internalization of professional meaning, rendering their job satisfaction less contingent on organizational support cues. Accordingly, the positive association between POS and job satisfaction is expected to be attenuated among teachers with high occupational commitment. Conversely, for teachers with lower occupational commitment—whose internal motivational resources may be comparatively limited—organizational support in the form of socio-emotional care, institutional assurances, and tangible resource provision becomes more pivotal for sustaining positive work experiences. Therefore, the positive relationship between POS and job satisfaction should be more pronounced when occupational commitment is low.

### 1.3 Research objectives

In summary, this study examines whether job satisfaction mediates the association between perceived organizational support and family–school collaboration, and whether occupational commitment moderates both the direct association and the indirect association via job satisfaction. By clarifying when and through what mechanism perceived organizational support translates into stronger family–school collaboration, the findings advance understanding of the psychological processes linking organizational resources to teachers’ collaborative practice.

## 2 Methods

### 2.1 Participants

A total of 1,312 participants were in-service kindergarten teachers recruited from public and private kindergartens in China. Convenience sampling was employed to invite in-service kindergarten teachers to participate in a questionnaire survey accessed via the platform Questionnaire Star.^1^ The sample consisted of 1,273 females, accounting for 97.00% of the participants, and 39 males, representing 3.00% of the total. With respect to participants’ majors, 1,259 (96.0%) reported majoring in preschool education or a related education field, whereas 53 (4.0%) reported other majors. Regarding weekly working hours, 254 (19.4%) worked ≤ 40 h, 641 (48.9%) worked 41–50 h, 272 (20.7%) worked 51–60 h, and 145 (11.1%) worked ≥ 60 h. Finally, in terms of kindergarten ownership/administration, 1,133 (86.4%) were employed in public kindergartens, 17 (1.3%) in collectively run kindergartens (publicly self-administered), 157 (12.0%) in non-subsidized private kindergartens (non-inclusive private), and 5 (0.4%) in collectively run kindergartens (commissioned management).

### 2.2 Measures

#### 2.2.1 Perceived organizational support

Perceived organizational support (POS; Ling et al., 2006) was utilized to assess Chinese teachers’ perceived support from their schools. The scale includes 24 items in 3 dimensions: 10 items about job-Related support (e.g., “When I perform well at work, my school takes notice”), 7 items about value recognition (e.g., “My school believes that retaining me is important to the school”), 7 items about benefit concern (e.g., My school would understand if I am occasionally absent due to personal reasons). Each item was rated on a 5-point Likert scale ranging from *1* (*strongly disagree*) to *5* (*strongly agree*). In this study, the overall scale’s internal consistency reliability coefficient was 0.971, and the Cronbach’s alpha coefficients for each dimension of job-Related support, value recognition, benefit concerns were 0.951, 0.939, and 0.933, respectively.

#### 2.2.2 Occupational commitment

Teachers’ occupational commitment was measured using the Chinese version of the Occupational commitment (OC) (Meyer et al., 1993), revised by Long and Li (2002) to fit the purpose of the study on Chinese teachers. The revised scale consists of 16 items which are divided into 3 dimensions: affective commitment (6 items, e.g., “I would be very happy to spend the rest of my career with this organization”), continuance commitment (5 items, e.g., “Right now, staying with my organization is a matter of necessity as much as desire”), and normative commitment (5 items, e.g., “This organization deserves my loyalty”). Each item was rated on a 5-point Likert scale ranging from 1 (*strongly disagree*) to 5 (*strongly agree*), with higher average scores indicating higher levels of occupational commitment. In this study, Cronbach’s alpha coefficient was 0.928.

#### 2.2.3 Job satisfaction

Teachers’ Job Satisfaction was measured using the Chinese version of the Job Satisfaction (JS) (Pepe et al., 2017). The revised scale consists of 9 items. (, e.g., “The quality of your relations with co-workers.”). Each item was rated on a 5-point Likert scale ranging from 1 (*strongly disagree*) to 5 (*strongly agree*), with higher average scores indicating higher levels of job satisfaction. In this study, Cronbach’s alpha coefficient was 0.953.

### 2.3 Family–school collaboration

The Family–School Collaboration (ERS) was used to measure Chinese teachers’ perceived level of family–school collaboration (Carlson et al., 2006). This scale contains 18 items and two subscales, namely, my involvement in my work (9 items, e.g., “Helps me to understand different viewpoints and this helps me be a better family member”), my involvement in my family (9 items, e.g., “Helps me to gain knowledge and this helps me be a better worker”), Each item was scored on a 5-point Likert scale (1 = *Strongly Disagree*, 6 = *agree*). In this study, the Cronbach’s alpha coefficient for the scale was 0.986 and the Cronbach’s alpha coefficients for each dimension of my involvement in my work, my involvement in my family were 0.971, 0.981, respectively.

### 2.4 Procedure

This study has been approved by the Ethics Committee of the author’s university. All participants signed written informed consent prior to data collection. In 2025, participants completed questionnaires on Perceived Organizational Support, Family–School Collaboration, Job Satisfaction, and Occupational Commitment through a commonly used online survey platform (see text footnote 1). Participants were informed of the anonymity and confidentiality of the survey throughout the study and were explicitly informed that they could withdraw voluntarily from the study at any stage without any adverse consequences.

### 2.5 Data analysis

First, descriptive statistics were conducted on the relevant variables, followed by Pearson correlation analysis to examine the relationships between the variables (see Table 1). Then, the PROCESS macro (Model 4) proposed by Hayes (2013) was used to examine the mediating role of Job Satisfaction between Perceived Organizational Support and Family–School Collaboration (see Table 2). In addition, PROCESS macro (Model 7) was used to explore the moderating role of Occupational Commitment in the direct and indirect relationship between Perceived Organizational Support and Family–School Collaboration (see Table 3). All models were tested for significance by the Bootstrap method with 5,000 resamplings to verify statistical significance of direct and indirect effects.

**Table 1:** Bivariate correlations among study variables.

Variable	*M*	*D*	1	2	3	4
Perceived organizational support	3.559	0.661	1			
Family–school collaboration	4.087	0.571	0.483**	1		
Job satisfaction	3.945	0.604	0.590**	0.663**	1	
Occupational commitment	3.747	0.615	0.735**	0.536**	0.597**	1

*p < 0.05, **p < 0.01, ***p < 0.001.

**Table 2:** Testing the mediation effect of perceived organizational support on family–school collaboration via job satisfaction.

Variables	Job satisfaction				Family–school collaboration				Family–school collaboration			
	*β*	*SE*	*LLCI*	*ULCI*	*β*	*SE*	*LLCI*	*ULCI*	*β*	*SE*	*LLCI*	*ULCI*
Perceived organizational support	0.589	0.022	0.546	0.634	0.142	0.025	0.092	0.191	0.483	0.024	0.436	0.531
Job satisfaction					0.579	0.025	0.530	0.629				
*R* ^2^	0.348				0.453				0.234			
*F*	699.132^***^				514.712^***^				399.548^***^			

*β* are standardized coefficients. SE, standard error; LLCI, lower limit of the 95% confidence interval; ULCI, upper limit of the 95% confidence interval. Each column is a regression model that predicts the criterion at the top of the column. *p < 0.05, ***p < 0.001.

**Table 3:** Testing the moderated mediation effect of perceived organizational support on family–school collaboration.

Variables	Model 1 (job satisfaction)			
	*β*	*SE*	*LLCI*	*ULCI*
Perceived organizational support	0.314	0.031	0.253	0.376
Occupational commitment	0.372	0.031	0.310	0.434
Perceived organizational support*	0.054	0.014	0.027	0.082
Occupational commitment				
*R^2^*	0.413			
*F*	306.796***			

*β* are standardized coefficients. SE, standard error; LLCI, lower limit of the 95% confidence interval; ULCI, upper limit of the 95% confidence interval. Each column is a regression model that predicts the criterion at the top of the column. *p < 0.05, **p < 0.01, ***p < 0.001.

## 3 Results

### 3.1 Bivariate correlation analysis

The results of the bivariate correlation analysis for the variables of interest are presented in Table 1. Perceived Organizational Support are positively associated with Family–School Collaboration (*r* = 0.483, *p* < 0.01), positively associated with Job Satisfaction (*r* = 0.590, *p* < 0.01), and positively associated with Occupational Commitment (*r* = 0.0735, *p* < 0.01). Family–School Collaboration exhibits a strong positive association with Job Satisfaction (*r* = 0.663, *p* < 0.01) and a statistically significant but positive association with Occupational Commitment (*r* = 0.0536, *p* < 0.05). Job Satisfaction exhibits a strong positive association with Occupational Commitment (*r* = 0.0597, *p* < 0.05). Therefore, Hypothesis 1 was supported.

### 3.2 Mediating effect of job satisfaction

**Figure 1:**
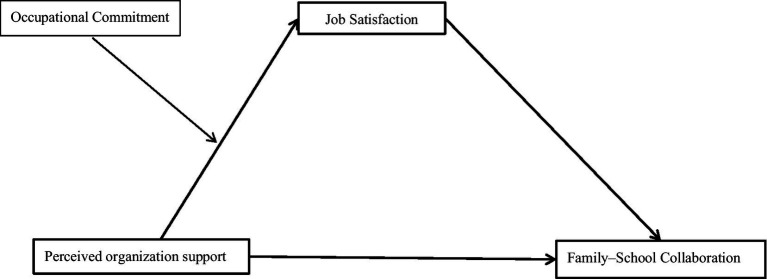
The assumed moderated mediation model.

Utilizing Model 4 of the PROCESS macro (Hayes, 2013), we investigated the mediating role of Job Satisfaction in the association between Perceived Organizational Support and Family–School Collaboration. As shown in Table 2, Perceived Organizational Support significantly positively predicted Job Satisfaction [*β* = 0.589, SE = 0.022, 95% CI (0.546, 0.634)] (Model 1). While controlling for perceived organizational support, job satisfaction was a significant predictor of family–school collaboration, *β* = 0.580, SE = 0.025, 95% CI [0.530, 0.629] (Model 2). The total effect of Perceived Organizational Support on Family–School Collaboration was also significant [*β* = 0.483, SE = 0.024, 95% CI (0.436, 0.531)] (Model 3). By bootstrapping 5,000 samples, the indirect effect of Perceived Organizational Support on Family–School Collaboration through Job Satisfaction was significant [ab = 0.342, SE = 0.031, 95%CI = (0.283, 0.405)], and the direct effect was also significant [c’ = 0.141, SE = 0.025, 95%CI = (0.092, 0.191)]. Thus, Job Satisfaction acts as a partial mediator in the relationship between Perceived Organizational Support and Family–School Collaboration, accounting for 70.81% of the total effect (see Figure 1).

### 3.3 Moderation effect of occupational commitment

**Figure 2:**
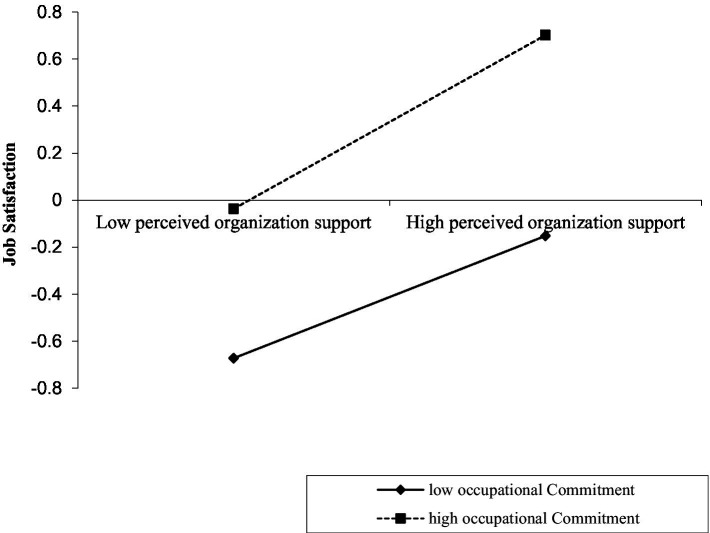
Occupational commitment moderates the indirect relationship between perceived organization support and job satisfaction.

We used Hayes’ (2013) PROCESS macro Model 7 to test a first-stage moderated mediation model in which occupational commitment moderated the association between perceived organizational support and job satisfaction, and job satisfaction subsequently predicted family–school collaboration. In the mediator model predicting job satisfaction, the overall model was significant [see Table 3, *R*^2^ = 0.413, *F*(3, 1,308) = 306.797, *p* < 0.001]. Perceived organizational support positively predicted job satisfaction [*β* = 0.315, *SE* = 0.031, *t* = 10.015, *p* < 0.001, 95% *CI* (0.253, 0.376)], and occupational commitment also showed a positive main effect [*β* = 0.372, *SE* = 0.032, *t* = 11.809, *p* < 0.001, 95% *CI* (0.310, 0.434)]. Importantly, the perceived organizational support × occupational commitment interaction was significant [*β* = 0.055, *SE* = 0.014, *t* = 3.938, *p* < 0.001, 95% *CI* (0.027, 0.082)], indicating that the positive association between perceived organizational support and job satisfaction was stronger at higher levels of occupational commitment. Simple slope tests showed that perceived organizational support more strongly predicted job satisfaction at high occupational commitment (+1 SD; *β* = 0.369, *t* = 11.678, *p <* 0.001) than at low occupational commitment (−1 SD; *β* = 0.260, *t* = 6.951, *p* < 0.001) (see Figure 2). In the outcome model predicting family–school collaboration, the overall model was significant, *R*^2^ = 0.453, *F*(2, 1,309) = 541.713, *p* < 0.001. Job satisfaction positively predicted family–school collaboration [*β* = 0.580, *SE* = 0.025, *t* = 22.897, *p* < 0.001, 95% *CI* (0.530, 0.629)], and perceived organizational support retained a significant direct effect [*β* = 0.142, *SE* = 0.025, *t* = 5.587, *p* < 0.001, 95% *CI* (0.092, 0.191)].

Bias-corrected bootstrap analyses (5,000 resamples) indicated that the conditional indirect effect of perceived organizational support on family–school collaboration via job satisfaction increased as occupational commitment increased: at −1 SD, indirect effect = 0.151 [Boot *SE* = 0.027, 95% Boot *CI* (0.101, 0.209)]; at the mean, indirect effect = 0.183 [Boot *SE* = 0.023, 95% Boot *CI* (0.139, 0.229)]; and at +1 SD, indirect effect = 0.214 [Boot *SE* = 0.026, 95% Boot *CI* (0.164, 0.266)]. The index of moderated mediation was significant [Index = 0.032, Boot *SE* = 0.013, 95% Boot CI (0.006, 0.055)], supporting moderated mediation.

## 4 Discussion

Although prior research has documented that perceived organizational support is significantly associated with family–school collaboration (Lin et al., 2025), comparatively little is known about the psychological mechanisms through which perceived organizational support shapes teachers’ collaboration with families. Focusing on kindergarten teachers, the present study examined (a) the mediating role of job satisfaction in the association between perceived organizational support and family–school collaboration and (b) the moderating role of occupational commitment in both the direct and indirect relationships between these variables. Pearson correlation analyses indicated that perceived organizational support was positively and significantly related to family–school collaboration. Mediation analyses further showed that job satisfaction partially mediated the relationship between perceived organizational support and family–school collaboration. Moreover, moderation analyses revealed that occupational commitment exerted significant moderating effects on both the direct and indirect pathways linking perceived organizational support to family–school collaboration. Specifically, when occupational commitment was higher, the positive association between perceived organizational support and job satisfaction was stronger. Collectively, these findings advance understanding of the internal mechanisms through which organizational support contributes to the quality of family–school collaboration in kindergarten contexts, thereby illuminating the psychological processes underlying “how” perceived organizational support translates into collaborative engagement.

### 4.1 Job satisfaction as a mediator

The present study found that job satisfaction mediated the association between perceived organizational support and family–school collaboration, thereby supporting our hypothesized mediating role of job satisfaction. This pattern suggests that perceived organizational support is not only directly linked to teachers’ job satisfaction but also indirectly related to family–school collaboration through the enhancement of job satisfaction.

Beyond the overall mediating effect, it is important to consider the specific indirect pathway underlying this process. First, perceived organizational support was positively associated with teachers’ job satisfaction; in other words, teachers who perceived higher levels of organizational support reported higher job satisfaction. This finding is consistent with prior evidence (Kiiza et al., 2025; Zhu et al., 2024). From the perspective of organizational support theory, when teachers perceive that their efforts are recognized and that the kindergarten provides institutional safeguards and socio-emotional care, they are more likely to form more favorable global evaluations of their work environment, which facilitates more satisfying and pleasurable work experiences. Moreover, the norm of reciprocity implies that, after receiving support, teachers may be more inclined to invest in their work in constructive ways and reciprocate toward the organization, further reinforcing satisfaction-related experiences (Gou et al., 2025; Zhu et al., 2024). Practically, these results indicate that kindergarten leadership should enhance the visibility, stability, and predictability of support by aligning institutional guarantees with consistent socio-emotional care, thereby fostering higher teacher job satisfaction.

Second, teachers’ job satisfaction was positively and significantly associated with family–school collaboration. This pattern is particularly meaningful in the kindergarten context, where “care–education integration” is a defining feature of daily practice. Kindergarten teachers must therefore maintain more frequent and fine-grained communication and coordination with parents, rendering family–school collaboration a work task characterized by high relational intensity and substantial emotional labor. Under such conditions, teachers’ positive affective evaluations of their work are likely to translate into sustained engagement in home–school interactions and higher-quality communication and coordination, thereby enhancing the overall effectiveness of family–school collaboration.

This finding can be interpreted through the broaden-and-build theory of positive emotions. The theory posits that positive emotions broaden individuals’ momentary thought–action repertoires by expanding attentional scope and cognitive processing, increasing behavioral flexibility, and, over time, building enduring psychological and social resources that support long-term adaptation and development (Roth et al., 2024; Seehagen et al., 2025). Accordingly, job satisfaction—as a relatively stable positive affective state—may broaden teachers’ attention and cognitive framing, strengthen psychological flexibility and problem-solving orientations in communication, and make them more inclined to respond empathically and communicate constructively, as well as to manage home–school differences in a cooperative manner (Peng, 2023; Wartenberg et al., 2023; Yotyodying et al., 2024). In addition, sustained positive interactions can facilitate the accumulation of relational resources (e.g., trust, mutual understanding, and cooperative norms), thereby improving both the interaction quality and collaborative effectiveness of kindergarten family–school partnerships (Fujisawa et al., 2025; Keung and Cheung, 2023).

### 4.2 Occupational commitment as a moderator

Occupational commitment moderated the association between perceived organizational support and teachers’ job satisfaction. Specifically, the present study further showed that, among kindergarten teachers, occupational commitment significantly strengthened the positive relationship between perceived organizational support and job satisfaction; that is, the positive association between perceived organizational support and job satisfaction was more pronounced for teachers with higher occupational commitment. This pattern can be interpreted through organizational support theory and social exchange theory (Kurt and Duyar, 2023; Liu et al., 2023; Zhang et al., 2023). Kindergarten teachers’ daily work typically entails dual responsibilities of care and education and is accompanied by substantial job demands, including heightened emotional labor, pressure associated with home–school communication, and safety-related accountability. In this context, teachers’ overall perceptions of the extent to which the kindergarten invests in resource provision, institutional support, and socio-emotional care constitute an important basis for evaluating the quality of their work environment (Cai et al., 2025; Liu et al., 2023; Zhang et al., 2023). When kindergarten teachers perceive higher organizational support, they are more likely to make reciprocity-based relational appraisals and develop more positive affective evaluations of their work, which, in turn, are associated with higher levels of job satisfaction. Moreover, organizational support serves both instrumental and symbolic functions; by meeting teachers’ social–emotional needs and reinforcing their sense of value and competence, it can enhance overall job evaluations and satisfaction (Kong et al., 2026; Li et al., 2026; Zhu et al., 2024). Occupational commitment may further amplify the beneficial effect of organizational support on job satisfaction. Compared with teachers with lower occupational commitment, highly committed teachers typically demonstrate stronger professional identification and more salient goal orientation toward their occupation. Prior research suggests that teachers with high occupational commitment tend to construe organizational support as a resource with both instrumental and relational value, which may strengthen perceived control and competence expectations, alleviate role-related strain, and foster organizational trust and expectations of relational stability (Chen, 2025; Kong et al., 2026; Zheng et al., 2025).

## 5 Limitations and future directions

First, design and inference constraints should be noted. The data were collected using a cross-sectional survey, which limits causal inference regarding the mediating pathway (POS → job satisfaction → family–school collaboration) and the conditional effects of occupational commitment. In addition, reliance on self-report measures may inflate associations due to shared method variance and perceptual consistency biases. Second, sampling and measurement scope restrict generalizability and construct coverage. The sample was drawn from kindergarten teachers, so the findings may not readily generalize to other educational stages, governance structures, or cultural settings. Moreover, family–school collaboration was assessed primarily from the teacher perspective; without parent- or administrator-reports, the results may not fully capture the dyadic and interactional nature of collaboration quality.

Future research could strengthen evidence by (a) adopting longitudinal or multi-wave designs to test temporal precedence and reduce alternative explanations; and (b) using multi-source and multi-method measurement to more accurately estimate collaboration outcomes and minimize common-method bias. In addition, it would be valuable to extend the model by examining multilevel and contextual mechanisms via multilevel SEM, and by testing additional boundary conditions to clarify for whom and under what conditions POS translates into higher job satisfaction and more effective family–school collaboration.

## Data Availability

The datasets presented in this study can be found in online repositories. The names of the repository/repositories and accession number(s) can be found in the article/supplementary material.

## Footnotes

1
www.wjx.cn
